# Supplementary figures and images for: Identifying latent subgroups of children with developmental delay using Bayesian sequential updating and Dirichlet process mixture modelling
Source: PLoS One. 2020 Jun 2;15(6):e0233542. doi: 10.1371/journal.pone.0233542 (PMC7266333; doi:10.1371/journal.pone.0233542)

## Group 1

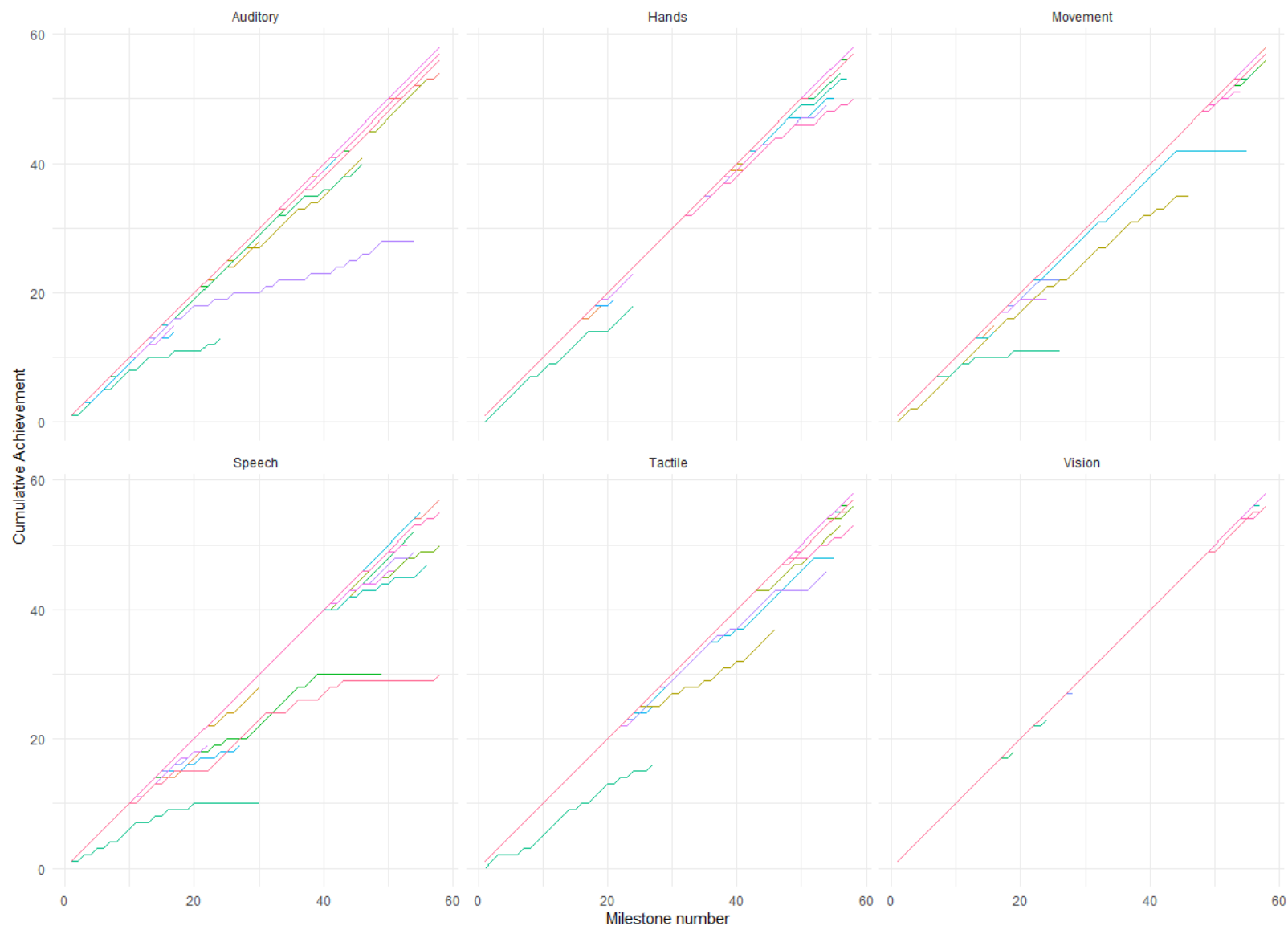

## Group 2

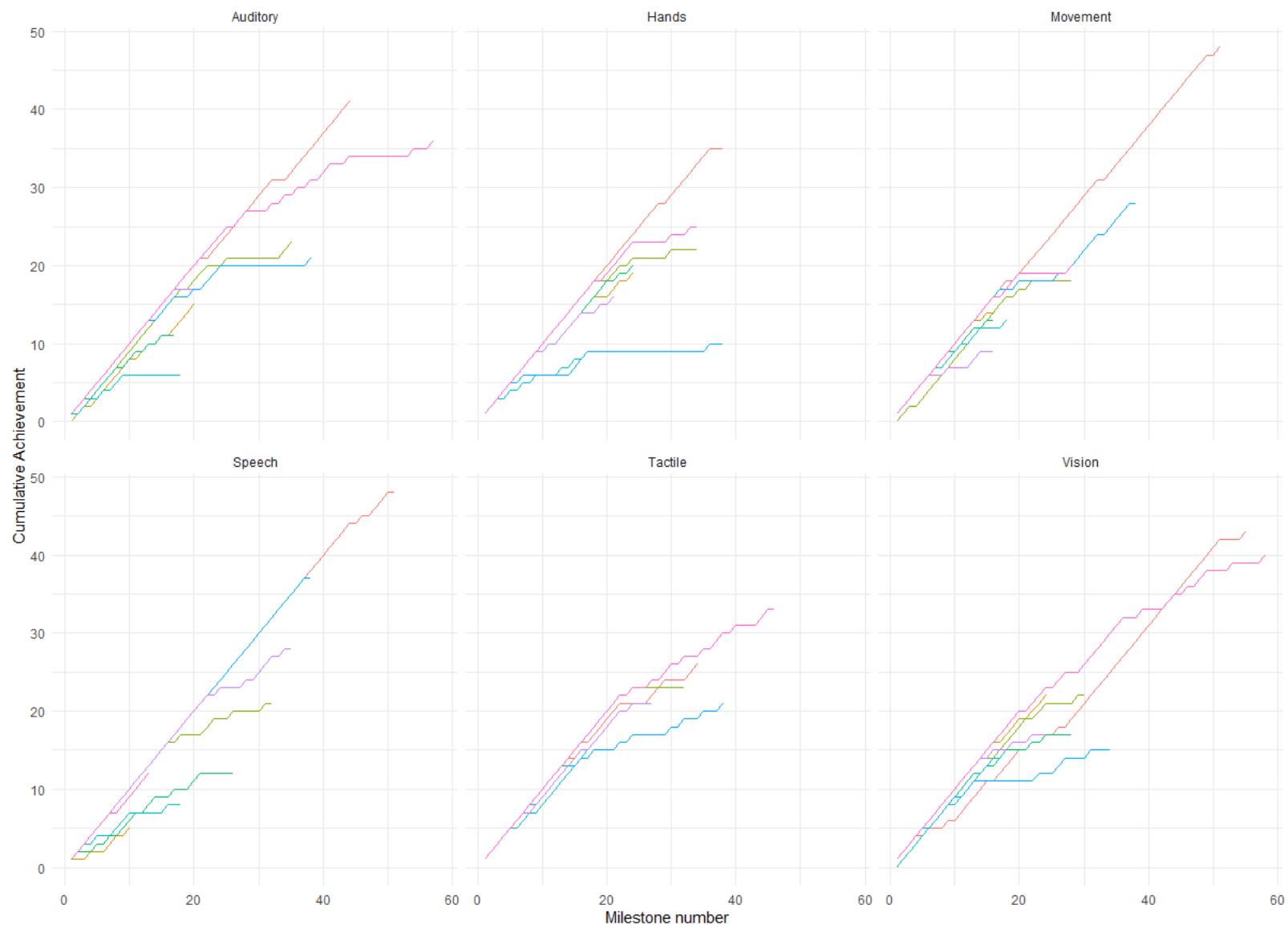

### Group 3

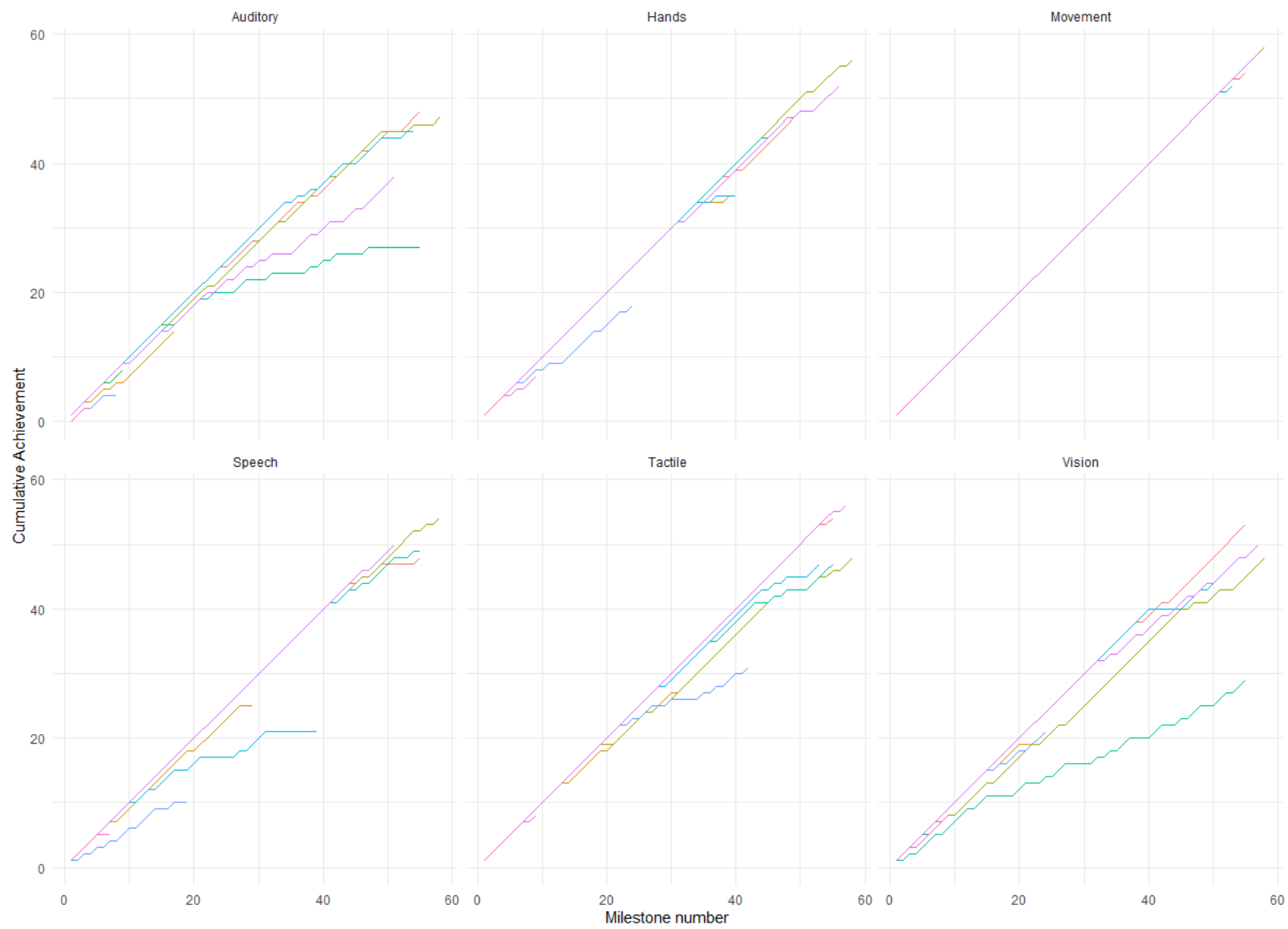

## Group 4

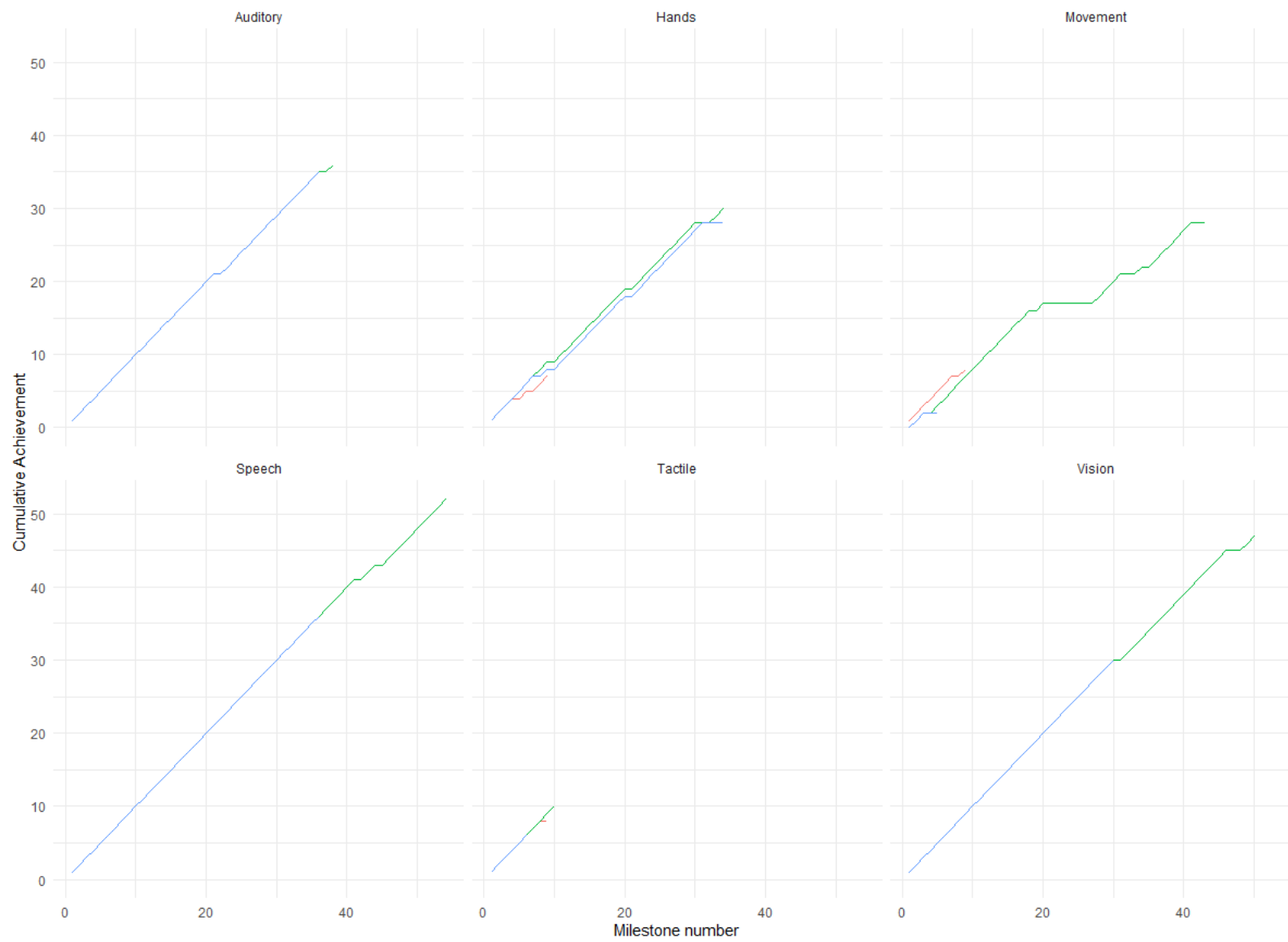

## Group 5

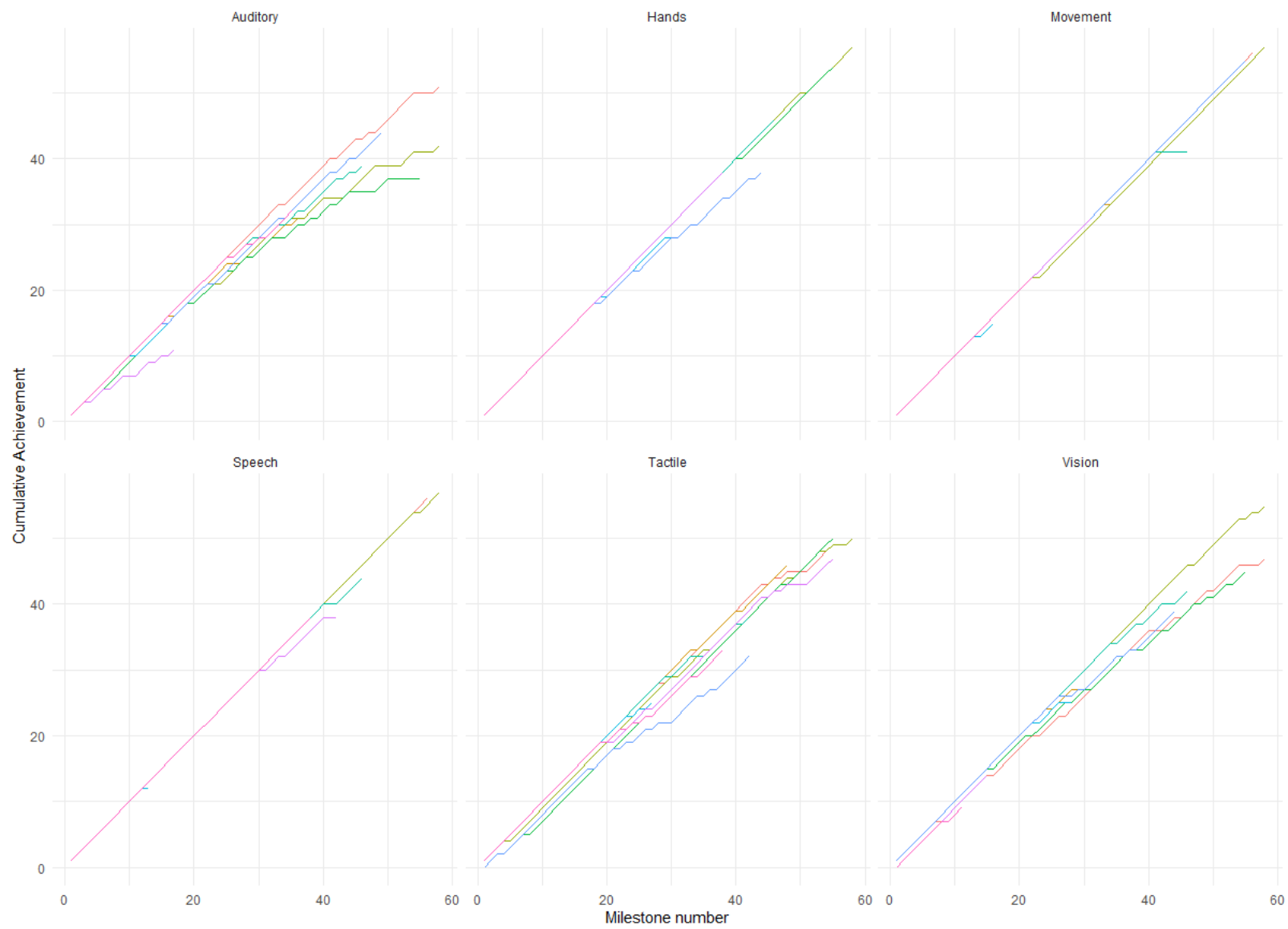

## Group 6

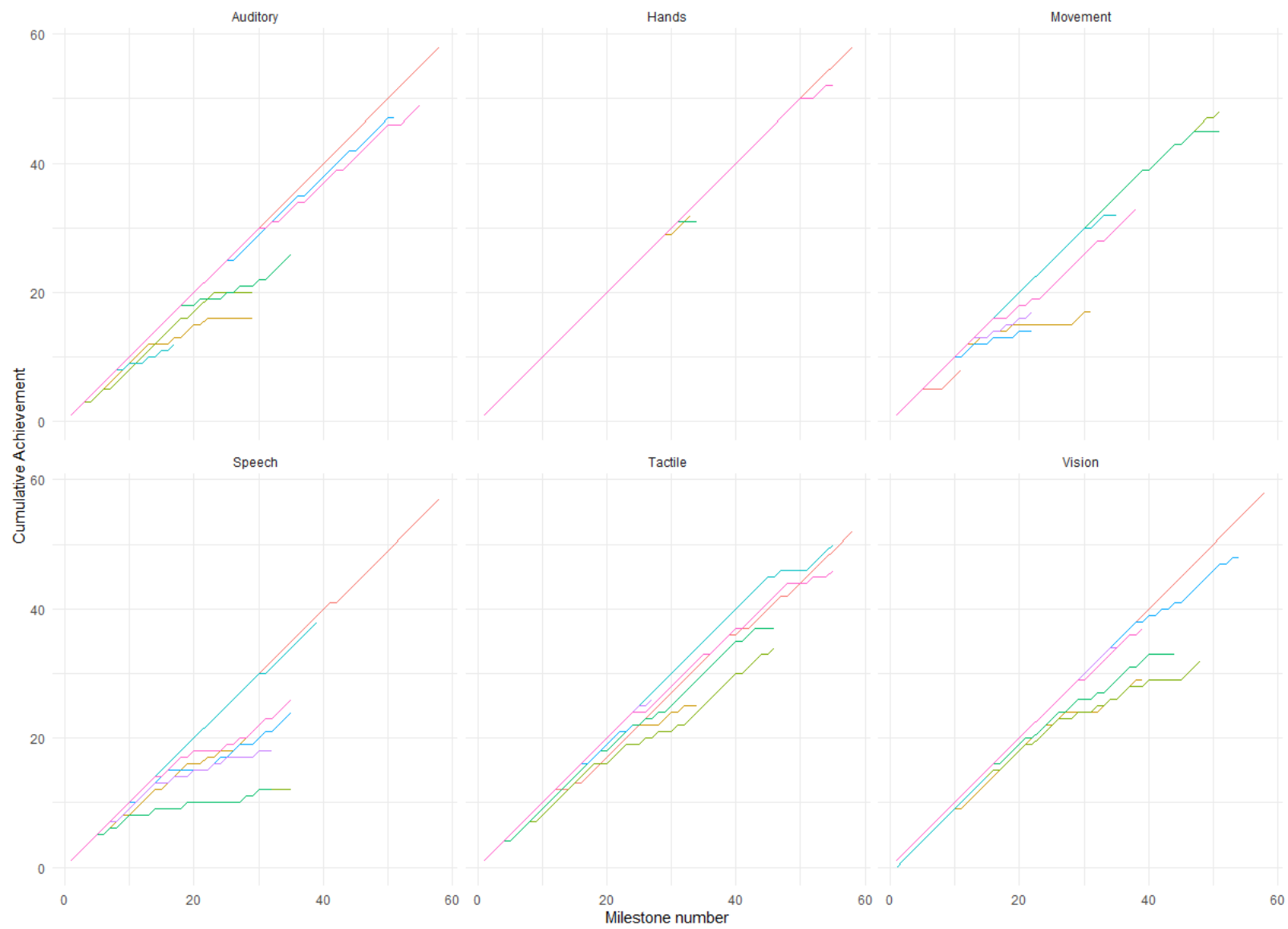

## Group 7

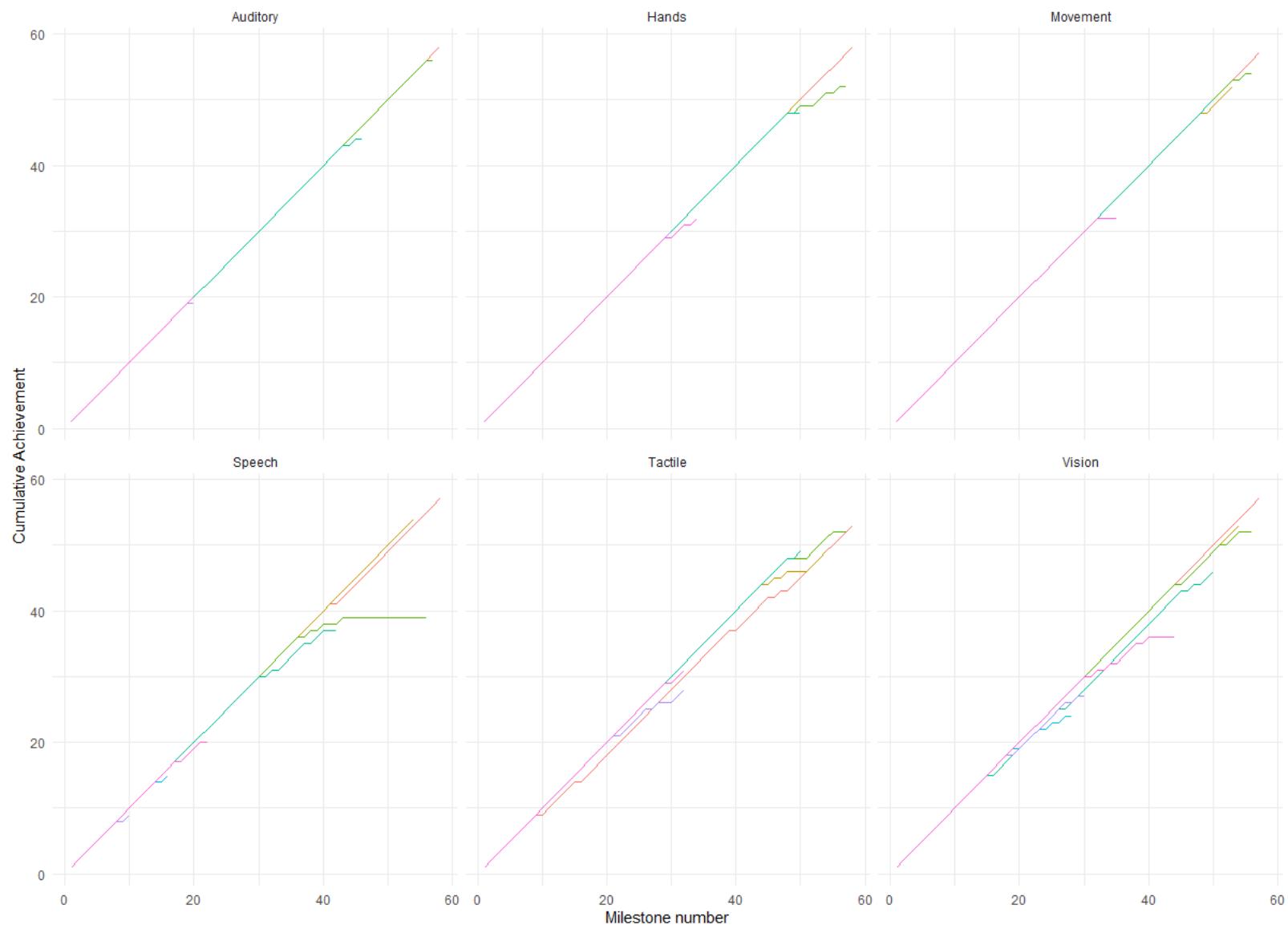

Group 8

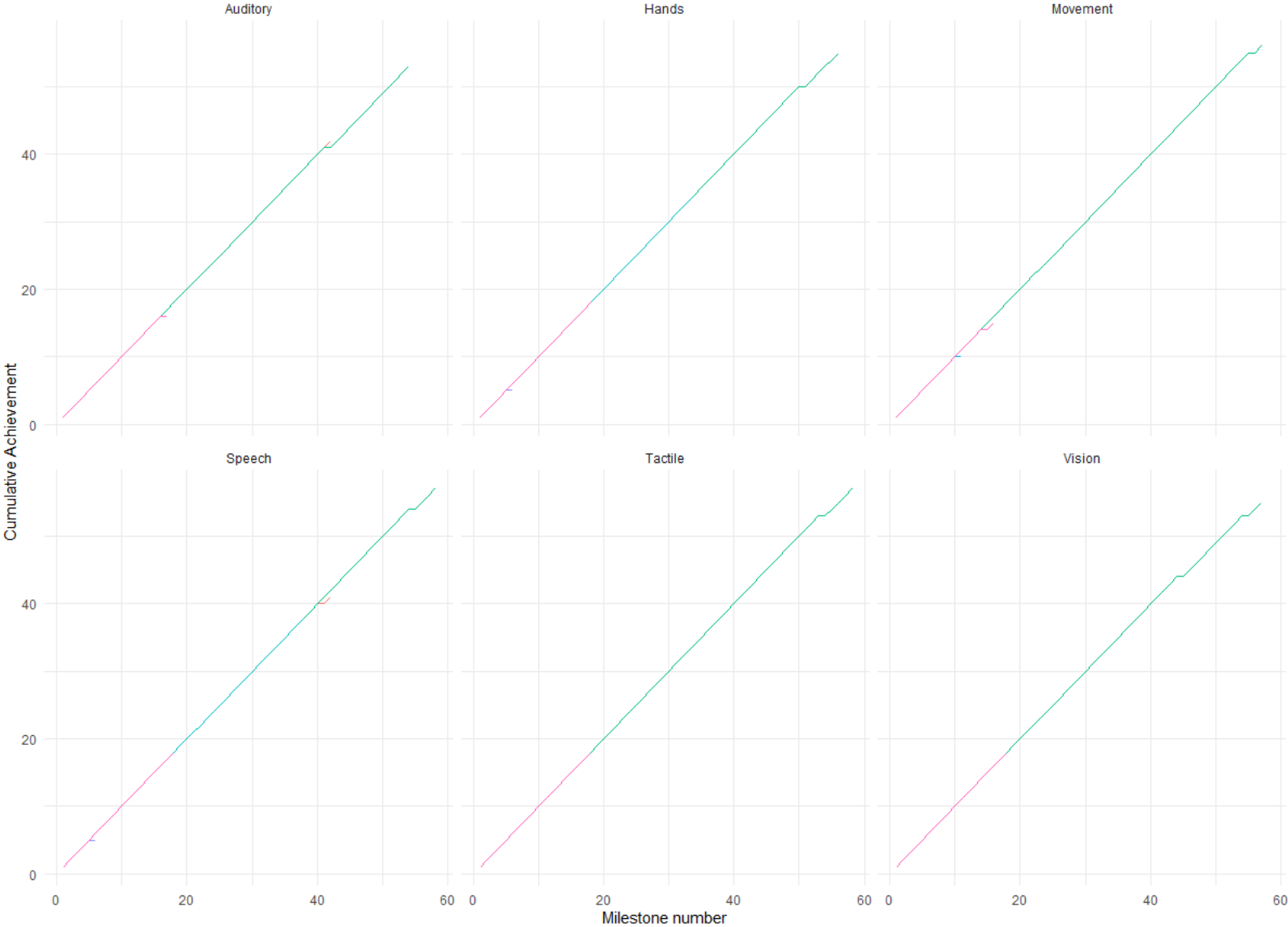

## Group 9

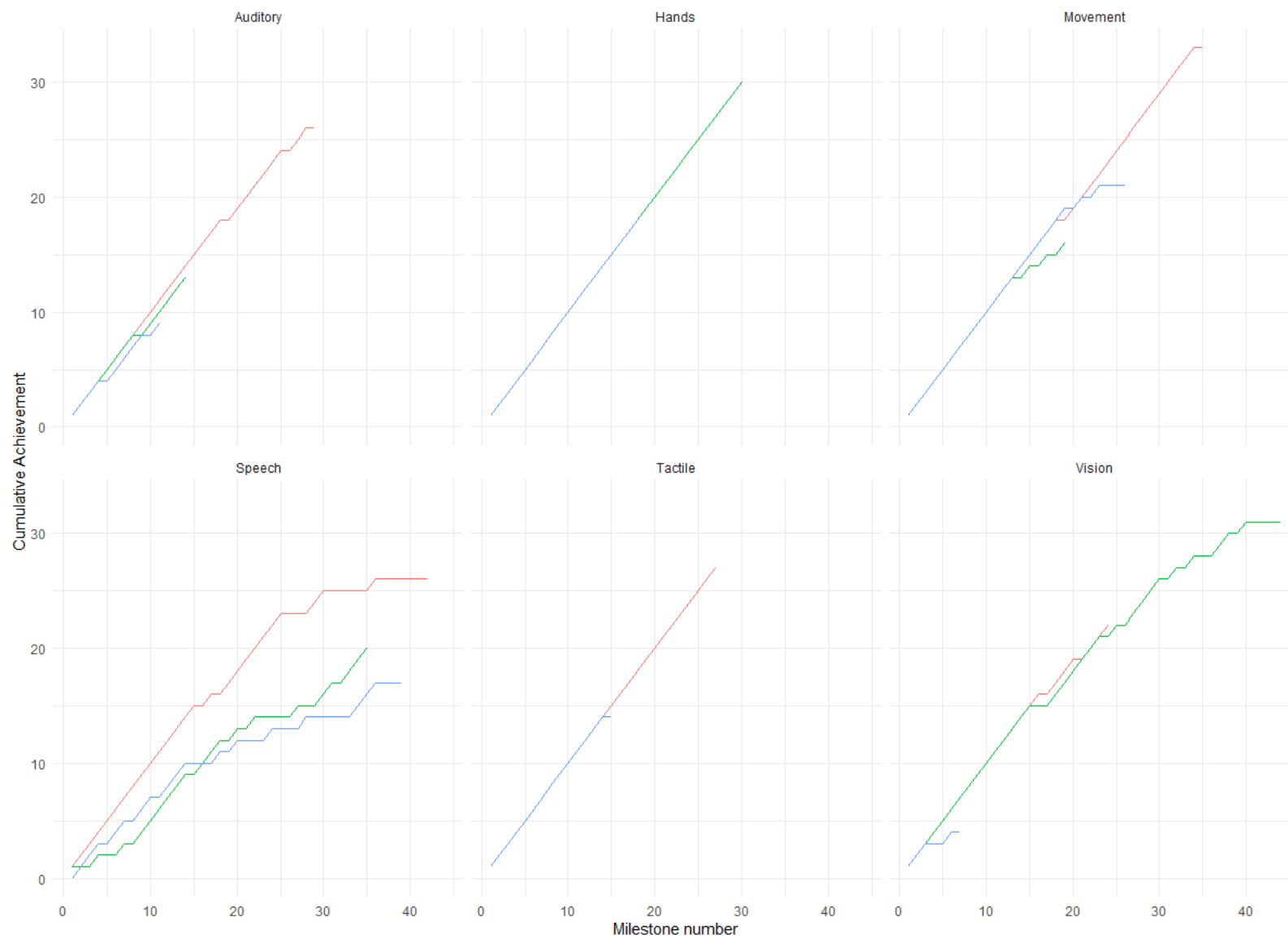

Supplement: S1 Fig — Cumulative sum of the achieved milestones for each functional domain for each group. (PDF) [file pone.0233542.s001.pdf]
