# Supplementary material for: Identifying latent subgroups of children with developmental delay using Bayesian sequential updating and Dirichlet process mixture modelling
Source: PLoS One. 2020 Jun 2;15(6):e0233542. doi: 10.1371/journal.pone.0233542 (PMC7266333; doi:10.1371/journal.pone.0233542)
Supplement: S1 Appendix — Appendix A: A brief summary of the Bayesian beta-Bernoulli model. Appendix B: A brief introduction to Dirichlet Process Mixture models. Appendix C: An overview of slice sampling. (PDF) [file pone.0233542.s002.pdf]

S1 Appendix. Additional model descriptions.

Appendix A: A brief summary of the Bayesian beta-Bernoulli model

Recall that for a single Bernoulli trial the likelihood of the data y given θ is

$$p(y|\theta) = \theta^y(1-\theta)^{1-y},$$

where $p(y|\theta) = \theta$ if $y = 1$ and $p(y|\theta) = (1-\theta)$ if $y = 0$. After observing y, the posterior probability for θ becomes

$$p(\theta|y) \propto \theta^y(1-\theta)^{1-y} \times p(\theta),$$

where $p(\theta)$ is the prior distribution for θ. The Beta distribution, which is conjugate to the Bernoulli distribution, can be used as a prior distribution for θ [1]. The density of the Beta distribution is given by

$$p(\theta|a,b) = \theta^{(a-1)}(1-\theta)^{(b-1)}/B(a,b),$$

where a and b are hyperparameters and $B(a,b)$ is a normalising constant [2]. When combining the Bernoulli likelihood function with the Beta prior distribution for a series of N independent trials with z successes, the posterior distribution for θ is again a Beta, given by

$$p(\theta|z,N) = \theta^{(z+a-1)}(1-\theta)^{(N-z+b-1)}/B(z+a, N-z+b).$$

The posterior mean for θ can be easily computed as $E(\theta|y) = \frac{z+a}{N+a+b}$ and the posterior variance can be computed as

$$var(\theta|y) = \frac{(a+z)(b+N-z)}{(a+b+N)^2(a+b+N+1)}.$$

Appendix B: A brief introduction to Dirichlet process mixture models

The Dirichlet process was first introduced by Ferguson [3] and is defined as a probability distribution over random probability measures [4]. The distribution of a Dirichlet process is (almost surely) discrete, in that a random sample drawn from a Dirichlet process has a nonzero probability that multiple draws will have identical values [5]. It is this discreteness property which makes the Dirichlet process ideal for clustering, as there is no need to specify the number of clusters *a priori* [6]. The basic Dirichlet process mixture model is formulated as follows:

$$\begin{aligned} y_i|\theta_i &\sim p(y_i|\theta_i) \\ \theta_i|G &\sim G \\ G &\sim DP(\alpha, G_0). \end{aligned}$$

The Dirichlet process models the distribution from which data $y_1, ..., y_n$ are drawn as a mixture of distributions, $p(y_i|\theta_i)$, where each parameter θ_i is drawn from a mixing distribution G [6]. This mixing distribution is given a Dirichlet process prior, with concentration parameter $\alpha > 0$ and base distribution G_0.

The base distribution is the prior expectation of G, i.e., $E[G] = G_0$, and the concentration parameter acts as an inverse variance where larger values of α result in smaller variances, which creates more concentrated draws around the mean of the base distribution [7].

Appendix C: An overview of the slice sampler

Slice sampling is an efficient adaptation of Gibbs sampling which can adapt easily to non-standard distributions [8]. The general premise is to introduce a latent variable u so that the joint density of y and u becomes

$$f_{C,\theta}(y, u) = \sum_k \mathbf{1}(u < C_k) N(y|\theta_k),$$

where u is uniformly distributed on the interval $(0, C_{k_i})$ [9]. Given u, the number of components is now finite, consisting of a subset indexed by $A_u = \{k : C_k > u\}$ [10]. The complete data likelihood for a sample $i = 1, ..., n$ is given by

$$l_{C,\theta}(y_i, u_i, z_i = k_i) = \prod_{i=1}^{n} \mathbf{1}(u_i < C_{k_i}) N(y_i|\theta_{k_i}),$$

where z is a variable identifying which cluster the observation y_i belongs to, which has the following conditional density:

$$p(z_i = k|...) \propto \mathbf{1}(k \in A_{u_i}) N(y_i|\theta_k).$$

The introduction of u means that only a finite set of stick weights, C_k, and corresponding parameters, θ_k need to be sampled at each iteration [9].

References

[1] Spiegelhalter DJ, Abrams KR, Myles JP. Bayesian approaches to clinical trials and health-care evaluation. vol. 13. John Wiley & Sons; 2004.

[2] Kruschke J. Doing Bayesian data analysis: A tutorial with R, JAGS, and Stan. Academic Press; 2014.

[3] Ferguson TS. A Bayesian analysis of some nonparametric problems. The annals of statistics. 1973; p. 209–230.

[4] Ghosal S. The Dirichlet process, related priors and posterior asymptotics. Bayesian nonparametrics. 2010;28:35.

[5] Green PJ, Richardson S. Modelling heterogeneity with and without the Dirichlet process. Scandinavian journal of statistics. 2001;28(2):355–375.

[6] Neal RM. Markov chain sampling methods for Dirichlet process mixture models. Journal of computational and graphical statistics. 2000;9(2):249–265.

[7] Teh YW. Dirichlet process. In: Encyclopedia of machine learning. Springer; 2011. p. 280–287.

[8] Neal RM. Slice sampling. Annals of statistics. 2003; p. 705–741.

[9] Walker SG. Sampling the Dirichlet mixture model with slices. Communications in StatisticsSimulation and Computation®. 2007;36(1):45–54.

[10] Kalli M, Griffin JE, Walker SG. Slice sampling mixture models. Statistics and computing. 2011;21(1):93–105.
