# Supplementary material for: Identifying latent subgroups of children with developmental delay using Bayesian sequential updating and Dirichlet process mixture modelling
Source: PLoS One. 2020 Jun 2;15(6):e0233542. doi: 10.1371/journal.pone.0233542 (PMC7266333; doi:10.1371/journal.pone.0233542)
Supplement: S2 Appendix — (PDF) [file pone.0233542.s003.pdf]

S2 Appendix. Grid experiment for selecting hyperparameters

A grid experiment was undertaken to test the performance of the DPMM for different hyperparameter specifications. The following hyperparameters were selected based on recommendations in the literature [1–4] and varied as follows. The precision parameter N_0 was set to $1, 0.5, 0.2, 0.1, 0.05$, or 0.01 [2]. The degrees of freedom for the inverse Wishart, c_0, was either $c_0 = r = 6$ or $c_0 = r + 1 = 7$, where r is equal to the number of covariates [4]. The scale parameter for the inverse Wishart, C_0, was selected as either the covariance matrix of the data Σ_y, $0.75\Sigma_y$ or $0.5\Sigma_y$ [1,4], and finally, the prior for the concentration parameter, α, was specified as either $Gamma(1, 1)$ or $Gamma(2, 2)$ [5, 6]. The vector of prior means $\mathbf{b}_0$ did not vary between models and was fixed at $\mathbf{b}_0 = \bar{\mathbf{y}}$. This resulted in 72 combinations of hyperparameters. The full list of hyperparameter combinations, as well as the effective sample size and autocorrelation statistics for the number of clusters, K, and concentration parameter, α, can be found in Table 1, Table 2 and Table 3 below.

The hyperparameter values chosen for the multivariate normal prior parameters (see Eq. 5 of the main text) were considered relatively uninformative while remaining within a sensible range for each parameter [7]. Similar hyperparameter specifications have been used in van Havre et al [7], Frühwirth-Schnatter [1] and Fraley and Rafferty [2]. The precision parameter N_0 is analogous to adding N_0 observations to each group in the data [2]. This parameter greatly influences the dispersion of the group means and, therefore, the values for this hyperparameter varied from small to large in order to compare and select the hyperparameter that would provide optimal dispersion. The hyperparameters for the prior on α were selected based on recommendations in the literature. The $Gamma(1, 1)$ prior was selected so that small values for α were more likely to be sampled, which results in the allocation of the data to fewer clusters [5]. The $Gamma(2, 2)$ prior was selected as it encourages both small and large values of α to be sampled [6].

The grid experiment was performed in two stages. In the first stage, each model ran for 100,000 iterations. Based on convergence statistics of the chains for the number of clusters, K, and α, 15 models that had the best effective sample size and autocorrelation for the different values of N_0 were selected (see Tables 1 to 3). The hyperparameters for the 15 selected models are displayed in Table 4. In the second stage, three chains for each of the 15 models were specified and run for 1,000,000 iterations each, to assess the long-term behaviour of the slice sampler. The three chains for each model were initialised using K-means, with the number of clusters specified as $K = 5$, $K = 10$ and $K = 15$, respectively.

Due to the small sample size and "noisy" (i.e., individuals do not group easily into clusters) data, the precision hyperparameter, N_0, had the largest effect on the performance of the slice sampler. For smaller values of N_0, there was larger dispersion in the group means, so the sampler would only sample a small number of clusters. This resulted in very little movement in the chain for

K, small effective sample sizes and high autocorrelation between iterations. For larger values of N_0, there was less dispersion, and more variation in the number of clusters sampled at each iteration, resulting in better convergence. However, this also resulted in a higher number of clusters to be sampled. The traceplots of α and K for the 15 selected models are available on Github [8]. The chains for each model were assessed for convergence using the Gelman-Rubin statistic [9]. Model convergence was achieved for 10 out of 15 models based on a Gelman-Rubin statistic of less than 1.1 for both K and α. The convergence statistics can be found in Table 5.

The chains for each model were combined by calculating the average posterior similarity matrix across the three chains. The PAM method was then used to select the optimal number of clusters for each model. The PAM procedure was implemented as follows: (1) The PAM algorithm was run on the pooled dissimilarity matrix for 2 to 20 clusters, (2) the average silhouette width for each clustering was recorded, (3) the scree plots of the average silhouette widths were inspected and (4) a clustering was selected that corresponded to the maximum average silhouette width prior to a dip or plateau in values on the scree plot. The scree plots for each model can be found on Github [8]. For some models, it was not immediately obvious which clustering to choose from the scree plot. For these models, the more parsimonious clustering was selected to avoid clusters of size $N = 1$. The average silhouette width of the selected number of clusters for each model is also displayed in Table 5.

Of the Models that had converged, Model 7 was selected as the final model. Although Model 3 had converged and had the highest average silhouette width, this model only returned 3 clusters, which was much fewer than the remaining converged models. It could be seen that the dispersion of the group means was too wide for this model, which was attributable to the small specification of the N_0 parameter. Of the remaining models, Model 7, Model 8 and Model 9 had the highest average silhouette widths. Of these models, Model 7 and Model 8 returned exactly the same clusters. The hyperparameters for these models only differed in terms of the specification for α, which did not appear to make any difference to the resulting clusters. Finally, there was only one difference in the obtained clusters between Model 9 and Model 7, where one cluster in Model 7 split to form two smaller clusters in Model 9. Ultimately, Model 7 was chosen over Model 9 as it had a larger average silhouette width, and was more parsimonious.

Table 1: Hyperparameters for all 72 models. Rows in bold indicate the 15 models selected for stage 2 of the grid experiment. Model 26 was selected for the final model(italicised).

Model	N_0	c_0	C_0	α
**1**	**0.01**	**6**	$\boldsymbol{\Sigma_y}$	$\boldsymbol{Gamma(1,1)}$
2	0.01	7	Σ_y	$Gamma(1,1)$
3	0.01	6	$0.75\Sigma_y$	$Gamma(1,1)$

Continued on next page

Table 1 – continued from previous page

Model	N_0	c_0	C_0	α
4	0.01	7	$0.75\Sigma_y$	$Gamma(1,1)$
5	0.01	6	$0.5\Sigma_y$	$Gamma(1,1)$
6	0.01	7	$0.5\Sigma_y$	$Gamma(1,1)$
7	0.01	6	Σ_y	$Gamma(2,2)$
8	0.01	7	Σ_y	$Gamma(2,2)$
9	0.01	6	$0.75\Sigma_y$	$Gamma(2,2)$
10	0.01	7	$0.75\Sigma_y$	$Gamma(2,2)$
11	0.01	6	$0.5\Sigma_y$	$Gamma(2,2)$
**12**	**0.01**	**7**	$\mathbf{0.5\Sigma_y}$	$\boldsymbol{Gamma(2,2)}$
13	0.05	6	Σ_y	$Gamma(1,1)$
**14**	**0.05**	**7**	$\boldsymbol{\Sigma_y}$	$\boldsymbol{Gamma(1,1)}$
15	0.05	6	$0.75\Sigma_y$	$Gamma(1,1)$
16	0.05	7	$0.75\Sigma_y$	$Gamma(1,1)$
17	0.05	6	$0.5\Sigma_y$	$Gamma(1,1)$
**18**	**0.05**	**7**	$\mathbf{0.5\Sigma_y}$	$\boldsymbol{Gamma(1,1)}$
**19**	**0.05**	**6**	$\boldsymbol{\Sigma_y}$	$\boldsymbol{Gamma(2,2)}$
20	0.05	7	Σ_y	$Gamma(2,2)$
21	0.05	6	$0.75\Sigma_y$	$Gamma(2,2)$
22	0.05	7	$0.75\Sigma_y$	$Gamma(2,2)$
23	0.05	6	$0.5\Sigma_y$	$Gamma(2,2)$
**24**	**0.05**	**7**	$\mathbf{0.5\Sigma_y}$	$\boldsymbol{Gamma(2,2)}$
25	0.10	6	Σ_y	$Gamma(1,1)$
26	***0.10***	***7***	$\boldsymbol{\Sigma_y}$	$\boldsymbol{Gamma(1,1)}$
27	0.10	6	$0.75\Sigma_y$	$Gamma(1,1)$
28	0.10	7	$0.75\Sigma_y$	$Gamma(1,1)$
29	0.10	6	$0.5\Sigma_y$	$Gamma(1,1)$
30	0.10	7	$0.5\Sigma_y$	$Gamma(1,1)$
31	0.10	6	Σ_y	$Gamma(2,2)$
**32**	**0.10**	**7**	$\boldsymbol{\Sigma_y}$	$\boldsymbol{Gamma(2,2)}$
33	0.10	6	$0.75\Sigma_y$	$Gamma(2,2)$
**34**	**0.10**	**7**	$\mathbf{0.75\Sigma_y}$	$\boldsymbol{Gamma(2,2)}$
35	0.10	6	$0.5\Sigma_y$	$Gamma(2,2)$
36	0.10	7	$0.5\Sigma_y$	$Gamma(2,2)$
37	0.20	6	Σ_y	$Gamma(1,1)$
38	0.20	7	Σ_y	$Gamma(1,1)$
39	0.20	6	$0.75\Sigma_y$	$Gamma(1,1)$
**40**	**0.20**	**7**	$\mathbf{0.75\Sigma_y}$	$\boldsymbol{Gamma(1,1)}$
41	0.20	6	$0.5\Sigma_y$	$Gamma(1,1)$
42	0.20	7	$0.5\Sigma_y$	$Gamma(1,1)$
43	0.20	6	Σ_y	$Gamma(2,2)$
**44**	**0.20**	**7**	$\boldsymbol{\Sigma_y}$	$\boldsymbol{Gamma(2,2)}$
45	0.20	6	$0.75\Sigma_y$	$Gamma(2,2)$
46	0.20	7	$0.75\Sigma_y$	$Gamma(2,2)$

Continued on next page

Table 1 – continued from previous page

Model	N_0	c_0	C_0	α
47	0.20	6	$0.5\Sigma_y$	$Gamma(2,2)$
48	0.20	7	$0.5\Sigma_y$	$Gamma(2,2)$
49	0.50	6	Σ_y	$Gamma(1,1)$
**50**	**0.50**	**7**	$\boldsymbol{\Sigma_y}$	$\boldsymbol{Gamma(1,1)}$
51	0.50	6	$0.75\Sigma_y$	$Gamma(1,1)$
52	0.50	7	$0.75\Sigma_y$	$Gamma(1,1)$
53	0.50	6	$0.5\Sigma_y$	$Gamma(1,1)$
54	0.50	7	$0.5\Sigma_y$	$Gamma(1,1)$
55	0.50	6	Σ_y	$Gamma(2,2)$
**56**	**0.50**	**7**	$\boldsymbol{\Sigma_y}$	$\boldsymbol{Gamma(2,2)}$
57	0.50	6	$0.75\Sigma_y$	$Gamma(2,2)$
58	0.50	7	$0.75\Sigma_y$	$Gamma(2,2)$
59	0.50	6	$0.5\Sigma_y$	$Gamma(2,2)$
60	0.50	7	$0.5\Sigma_y$	$Gamma(2,2)$
61	1.00	6	Σ_y	$Gamma(1,1)$
**62**	**1.00**	**7**	$\boldsymbol{\Sigma_y}$	$\boldsymbol{Gamma(1,1)}$
63	1.00	6	$0.75\Sigma_y$	$Gamma(1,1)$
64	1.00	7	$0.75\Sigma_y$	$Gamma(1,1)$
65	1.00	6	$0.5\Sigma_y$	$Gamma(1,1)$
66	1.00	7	$0.5\Sigma_y$	$Gamma(1,1)$
67	1.00	6	Σ_y	$Gamma(2,2)$
**68**	**1.00**	**7**	$\boldsymbol{\Sigma_y}$	$\boldsymbol{Gamma(2,2)}$
69	1.00	6	$0.75\Sigma_y$	$Gamma(2,2)$
70	1.00	7	$0.75\Sigma_y$	$Gamma(2,2)$
71	1.00	6	$0.5\Sigma_y$	$Gamma(2,2)$
72	1.00	7	$0.5\Sigma_y$	$Gamma(2,2)$

Table 2: Effective sample size (ESS) and autocorrelation (AC) for K for all 72 models after 100,000 iterations. Rows in bold indicate the 15 models selected for stage 2 of the grid experiment. Model 26 was chosen as the final model (italicised).

Model	ESS	AC lag 1	AC lag 5	AC lag 10	AC lag 50
**1**	**23.73**	**0.999**	**0.996**	**0.993**	**0.972**
2	15.50	0.999	0.998	0.996	0.984
3	12.32	0.999	0.998	0.997	0.987
4	6.31	0.999	0.999	0.999	0.994
5	5.32	0.999	0.999	0.998	0.994
6	1.68	0.999	0.999	0.999	0.998
7	15.09	0.999	0.998	0.996	0.985
8	9.02	0.999	0.999	0.998	0.991
9	10.94	0.999	0.998	0.997	0.987
10	3.77	0.999	0.999	0.999	0.996
11	1.79	0.999	0.999	0.999	0.998
**12**	**19.17**	**0.999**	**0.998**	**0.996**	**0.980**
13	31.41	0.998	0.994	0.989	0.963
**14**	**46.76**	**0.998**	**0.991**	**0.985**	**0.946**
15	13.40	0.999	0.996	0.994	0.980
16	7.25	0.999	0.998	0.997	0.991
17			non-convergence		
**18**	**72.57**	**0.997**	**0.989**	**0.980**	**0.926**
19	**36.32**	**0.998**	**0.992**	**0.987**	**0.954**
20	13.19	0.999	0.997	0.995	0.983
21	9.49	0.999	0.998	0.997	0.989
22	12.03	0.999	0.997	0.996	0.985
23	14.37	0.999	0.997	0.995	0.983
**24**	**74.60**	**0.997**	**0.989**	**0.980**	**0.925**
25	19.80	0.998	0.994	0.991	0.974
26	***181.87***	***0.990***	***0.962***	***0.936***	***0.804***
27	22.09	0.998	0.994	0.990	0.971
28	173.26	0.992	0.968	0.944	0.819
29			non-convergence		
30	146.49	0.994	0.976	0.956	0.847
31	26.82	0.998	0.993	0.988	0.964
**32**	**150.78**	**0.992**	**0.968**	**0.944**	**0.827**
33	32.35	0.997	0.991	0.985	0.956
**34**	**153.33**	**0.992**	**0.969**	**0.946**	**0.824**
35			non-convergence		
36	148.84	0.995	0.979	0.962	0.861
37	67.71	0.994	0.980	0.968	0.913
38	327.24	0.984	0.937	0.895	0.699
					Continued on next page

Table 2 – continued from previous page

Model	ESS	AC lag 1	AC lag 5	AC lag 10	AC lag 50
39			non-convergence		
**40**	**374.32**	**0.984**	**0.934**	**0.887**	**0.665**
41	164.06	0.990	0.961	0.933	0.808
42			non-convergence		
43	56.12	0.995	0.983	0.973	0.926
**44**	**362.99**	**0.980**	**0.924**	**0.873**	**0.650**
45	109.24	0.992	0.971	0.952	0.867
46	322.05	0.984	0.935	0.891	0.687
47			non-convergence		
48			non-convergence		
49	515.52	0.968	0.890	0.827	0.574
**50**	**704.41**	**0.964**	**0.873**	**0.796**	**0.483**
51	523.86	0.973	0.903	0.842	0.579
52	619.48	0.970	0.890	0.820	0.517
53	343.68	0.980	0.922	0.871	0.653
54	356.18	0.981	0.927	0.877	0.666
55	518.78	0.969	0.892	0.828	0.565
**56**	**674.10**	**0.966**	**0.879**	**0.805**	**0.501**
57	432.51	0.975	0.910	0.855	0.622
58	563.02	0.971	0.894	0.827	0.543
59			non-convergence		
60			non-convergence		
61	662.02	0.961	0.871	0.799	0.507
**62**	**929.18**	**0.953**	**0.840**	**0.747**	**0.390**
63	565.91	0.965	0.876	0.804	0.520
64	798.95	0.961	0.865	0.782	0.455
65	437.72	0.975	0.909	0.853	0.610
66	439.76	0.975	0.908	0.848	0.599
67	773.98	0.959	0.865	0.789	0.471
**68**	**1015.70**	**0.954**	**0.841**	**0.751**	**0.384**
69	628.91	0.965	0.880	0.810	0.524
70	749.55	0.961	0.862	0.779	0.463
71	438.27	0.975	0.909	0.854	0.618
72	364.87	0.977	0.913	0.858	0.635

Table 3: Effective sample size (ESS) and autocorrelation (AC) for α for all 72 models after 100,000 iterations. Rows in bold indicate the 15 models selected for stage 2 of the grid experiment. Model 26 was chosen as the final model (italicised).

Model	ESS	AC lag 1	AC lag 5	AC lag 10	AC lag 50
**1**	**1308.17**	**0.154**	**0.153**	**0.152**	**0.147**
2	476.90	0.251	0.247	0.246	0.242
3	281.09	0.303	0.309	0.303	0.307
4	95.36	0.465	0.463	0.460	0.464
5	98.93	0.463	0.465	0.463	0.463
6	53.00	0.563	0.559	0.561	0.561
7	331.68	0.293	0.290	0.294	0.201
8	104.80	0.468	0.466	0.463	0.460
9	361.13	0.273	0.271	0.276	0.268
10	67.66	0.522	0.522	0.522	0.521
11	51.01	0.578	0.577	0.577	0.574
**12**	**579.02**	**0.224**	**0.230**	**0.225**	**0.225**
13	409.02	0.272	0.269	0.276	0.268
**14**	**621.57**	**0.239**	**0.239**	**0.240**	**0.228**
15	147.58	0.423	0.419	0.417	0.415
16	32.71	0.714	0.712	0.712	0.705
17			non-convergence		
**18**	**599.43**	**0.259**	**0.260**	**0.256**	**0.239**
**19**	**876.39**	**0.188**	**0.192**	**0.190**	**0.187**
20	88.23	0.518	0.512	0.514	0.508
21	68.80	0.544	0.540	0.540	0.540
22	57.82	0.597	0.596	0.593	0.589
23	124.26	0.473	0.474	0.469	0.462
**24**	**547.43**	**0.246**	**0.266**	**0.263**	**0.248**
25	94.49	0.539	0.539	0.539	0.526
26	***1251.31***	***0.219***	***0.217***	***0.208***	***0.179***
27	99.86	0.536	0.535	0.531	0.521
28	843.01	0.273	0.271	0.264	0.227
29			non-convergence		
30	603.25	0.323	0.319	0.312	0.275
31	148.97	0.459	0.459	0.455	0.422
**32**	**925.98**	**0.257**	**0.255**	**0.244**	**0.216**
33	188.56	0.427	0.429	0.422	0.412
**34**	**871.14**	**0.276**	**0.273**	**0.264**	**0.230**
35			non-convergence		
36	510.77	0.350	0.345	0.336	0.303
37	280.36	0.439	0.430	0.425	0.393
38	1191.52	0.295	0.288	0.270	0.209

Continued on next page

Table 3 – continued from previous page

Model	ESS	AC lag 1	AC lag 5	AC lag 10	AC lag 50
39			non-convergence		
**40**	**1328.22**	**0.304**	**0.293**	**0.276**	**0.203**
41	846.44	0.302	0.293	0.285	0.239
42			non-convergence		
43	219.82	0.468	0.462	0.455	0.429
**44**	**1558.80**	**0.270**	**0.260**	**0.242**	**0.182**
45	453.65	0.375	0.367	0.359	0.326
46	1250.88	0.298	0.287	0.279	0.208
47			non-convergence		
48			non-convergence		
49	2044.40	0.259	0.242	0.222	0.154
**50**	**2283.61**	**0.302**	**0.282**	**0.256**	**0.149**
51	1694.30	0.282	0.272	0.245	0.161
52	1963.11	0.310	0.297	0.272	0.168
53	1491.91	0.291	0.278	0.263	0.191
54	1159.67	0.337	0.320	0.305	0.228
55	2104.50	0.263	0.244	0.224	0. 146
**56**	**2136.88**	**0.315**	**0.295**	**0.268**	**0.162**
57	1663.99	0.290	0.278	0.258	0.183
58	1770.63	0.322	0.303	0.277	0.177
59			non-convergence		
60			non-convergence		
61	2304.06	0.285	0.268	0.244	0.148
**62**	**3020.09**	**0.300**	**0.272**	**0.240**	**0.118**
63	2143.54	0.289	0.267	0.242	0.157
64	2505.92	0.301	0.281	0.247	0.142
65	1556.50	0.313	0.291	0.276	0.193
66	1495.52	0.320	0.304	0.286	0.199
67	2586.93	0.288	0.269	0.237	0.140
**68**	**3260.57**	**0.303**	**0.275**	**0.242**	**0.118**
69	2059.52	0.298	0.275	0.260	0.160
70	2570.59	0.294	0.270	0.244	0.142
71	1453.71	0.313	0.301	0.275	0.200
72	1283.49	0.334	0.316	0.294	0.218

Table 4: Hyperparameters and the number of obtained clusters for the 15 selected models. The final selected model is italicised.

Model	N_0	c_0	C_0	α
1	0.01	6	Σ_y	Gamma(1,1)
2	0.01	7	$0.5\Sigma_y$	Gamma(2,2)
3	0.05	7	Σ_y	Gamma(1,1)
4	0.05	7	$0.5\Sigma_y$	Gamma(1,1)
5	0.05	6	Σ_y	Gamma(2,2)
6	0.05	7	$0.5\Sigma_y$	Gamma(2,2)
7	*0.10*	*7*	Σ_y	*Gamma(1,1)*
8	0.10	7	Σ_y	Gamma(2,2)
9	0.10	7	$0.75\Sigma_y$	Gamma(2,2)
10	0.20	7	$0.75\Sigma_y$	Gamma(1,1)
11	0.20	7	Σ_y	Gamma(2,2)
12	0.50	7	Σ_y	Gamma(1,1)
13	0.50	7	Σ_y	Gamma(2,2)
14	1.00	7	Σ_y	Gamma(1,1)
15	1.00	7	Σ_y	Gamma(2,2)

Table 5: Gelman Rubin (GR) statistic for K and α, the average silhouette width (ASW) of the optimal number of clusters using the PAM method for the combined chains of each converged model. The PAM method was not performed for models that did not converge. The final selected model is italicised.

Model	GR K	GR α	ASW	No. of clusters
1	1.25	1.00	-	-
2	2.42	1.05	-	-
3	1.02	1.00	0.851	3
4	2.56	2.00	-	-
5	1.15	1.01	-	-
6	1.59	1.43	-	-
7	*1.01*	*1.00*	*0.452*	*9*
8	1.10	1.02	0.449	9
9	1.00	1.00	0.448	10
10	1.00	1.00	0.406	13
11	1.00	1.00	0.425	12
12	1.00	1.00	0.358	11
13	1.00	1.00	0.358	11
14	1.00	1.00	0.326	16
15	1.00	1.00	0.327	16

References

[1] Frühwirth-Schnatter S. Finite mixture and Markov switching models. Springer Science & Business Media; 2006.

[2] Fraley C, Raftery AE. Bayesian regularization for normal mixture estimation and model-based clustering. Journal of classification. 2007;24(2):155–181.

[3] Schaefer J. Analysis of incomplete multivariate data by simulation. Chapmann and Hall, London. 1997;.

[4] Schuurman N, Grasman R, Hamaker E. A comparison of inverse-wishart prior specifications for covariance matrices in multilevel autoregressive models. Multivariate Behavioral Research. 2016;51(2-3):185–206.

[5] Gelman A, Stern HS, Carlin JB, Dunson DB, Vehtari A, Rubin DB. Bayesian data analysis. Chapman and Hall/CRC; 2013.

[6] Ishwaran H, James LF. Approximate Dirichlet process computing in finite normal mixtures: smoothing and prior information. Journal of Computational and Graphical statistics. 2002;11(3):508–532.

[7] van Havre Z, White N, Rousseau J, Mengersen K. Clustering action potential spikes: Insights on the use of overfitted finite mixture models and Dirichlet process mixture models. arXiv preprint arXiv:160201915. 2016;.

[8] Gilholm P. Bayesian Sequential Updating DPMM supplementary; 2019. Available from: `https://github.com/TrishG89/Bayesian_Sequential_Updating_DPMM_supplementary`.

[9] Gelman A, Rubin DB, et al. Inference from iterative simulation using multiple sequences. Statistical science. 1992;7(4):457–472.
