# Supplementary material for: Identifying latent subgroups of children with developmental delay using Bayesian sequential updating and Dirichlet process mixture modelling
Source: PLoS One. 2020 Jun 2;15(6):e0233542. doi: 10.1371/journal.pone.0233542 (PMC7266333; doi:10.1371/journal.pone.0233542)
Supplement: S3 Appendix — (PDF) [file pone.0233542.s004.pdf]

S3 Appendix. Comparison of DPMM to K-means and model-based clustering.

K-means and model-based clustering were used to cluster the areas obtained from the Bayesian sequential updating. Brief descriptions of these methods are provided, followed by the results of the clustering for each method. The R statistical computing software [1] was used to fit all the models. Finally, the clusters obtained through these alternative methods are compared to the clusters obtained from the DPMM.

K-means

The K-means algorithm [2] is a non-parametric unsupervised clustering method which assigns each observation to one of K pre-specified clusters [3]. Each observation is assigned to a cluster so that the within-cluster variation is minimized. The algorithm has the following steps:

1. Each observation is initially randomly assigned to one of K clusters.
2. The cluster centroids (i.e., vector of p-variable means) are calculated for each cluster.
3. The Euclidean distance is calculated between the observation and each cluster centroid, and the observation is assigned to the cluster with the smallest Euclidean distance as follows

$$C(i) = \underset{1 \leq k \leq K}{\arg\min} ||x_i - m_k||^2, \quad (1)$$

   where m_k is the cluster centroid for cluster k [4].

4. steps (2) and (3) are repeated until observations are no longer reassigned to clusters.

As K-means requires the user to pre-specify the number of clusters prior to the analysis, the same approach was used for selecting the optimal number of clusters as was used with the PAM method for the DPMM. That is, the average silhoutte width was calculated for the number of clusters ranging from 2 to 20 and a clustering was selected which maximises the average silhouette width (refer to paper for details).

Results

The K-means algorithm returned two clusters as the optimal number, as determined by the maximum average silhouette width. The scree plot of the average silhouette width for 2 to 20 clusters is displayed in Figure 1. The descriptive statistics for the two clusters are displayed in Table 1 and the cluster profiles for the two groups are displayed in Figure 2.

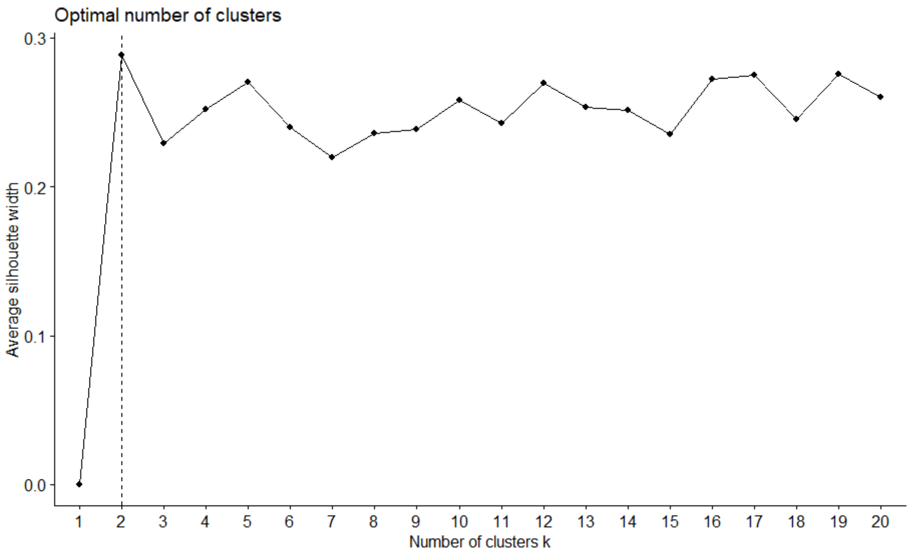

Figure 1: Average silhouette width for 2 to 20 clusters using the K-means algorithm.

Table 1: Cluster sizes, means and standard deviations for the two clusters obtained from the K-means algorithm

Cluster	Size	Domain	Mean	Standard Deviation
1	46	Auditory	0.016	0.027
		Hands	0.007	0.079
		Movement	0.012	0.034
		Speech	0.023	0.049
		Tactile	0.008	0.018
		Vision	0.011	0.024
2	30	Auditory	0.129	0.091
		Hands	0.057	0.101
		Movement	0.058	0.076
		Speech	0.144	0.164
		Tactile	0.074	0.076
		Vision	0.089	0.091

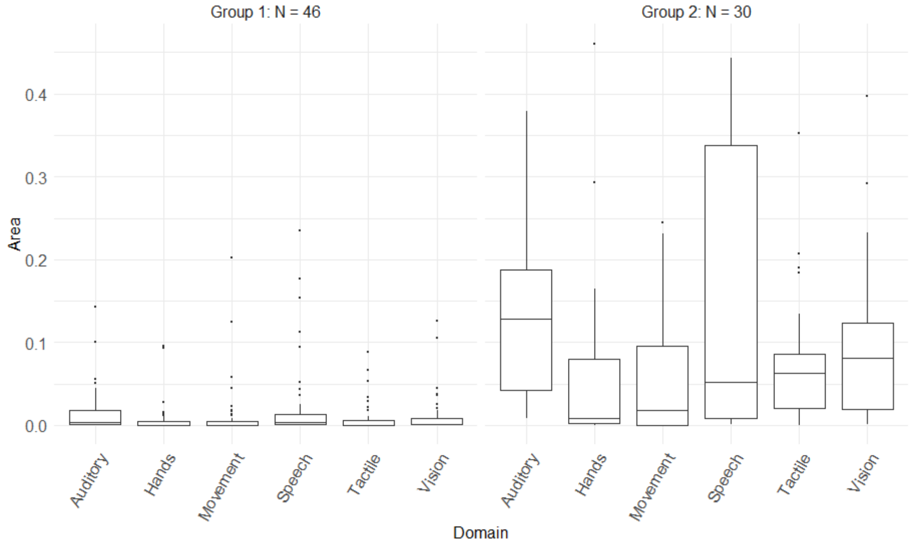

Figure 2: Cluster profiles of the two groups obtained using the K-means algorithm.

It can be seen from Figure 2 that Group 1 mainly consists of children who are developing typically across the functional domains, with areas close to zero. However, there are a large number of outlying points for each domain, indicating that not all children fit this profile of typical development for all domains. The second group consists of 30 children who are displaying moderate to severe departures from typical development for all domains. Once again, due to the variability in the widths of the box plots, one cluster indicating atypical development for all domains may not be sufficient to capture the differences in development for these children.

Model-based clustering

Model-based clustering models the density of a sample of n observations $x = \{x_1, ..., x_i, ..., x_n\}$ as a finite mixture of G components as follows:

$$f(y_i) = \sum_{k=1}^{G} \pi_k f_k(y_i|\theta_k) \tag{2}$$

where θ_k are the parameters of the mixture model and π_k are the mixture weights, such that $\pi_k > 0, \sum_{k=1}^{G} \pi_k = 1$ [5]. When G is fixed, the parameters π and θ are unknown and need to be estimated. In order to estimate these parameters, a latent component-indicator $z = \{z_1, ...z_n\}$ is introduced where each z_i is a K-dimensional vector, where the kth element of z_i takes the value 1

if y_i comes from component k ($k = 1, ..., G$) and takes the value 0, otherwise [6]. Most often, the estimation of the parameters of a mixture model is accomplished through the use of the Expectation-Maximisation (EM) algorithm which iterates between two steps [6]. The first step (expectation) takes the conditional expectation of the complete-data log-likelihood, given the observed data y and the current estimate of the parameters θ [6]. The second step (maximisation) maximises the log-likelihood with respect to θ, to give the updated parameter estimates [6]. The algorithm iterates between these two steps until convergence.

For this implementation of model-based clustering, consistent with the DPMM, it is assumed that each component follows a multivariate normal distribution, i.e., $f_k(y) \sim MVN(\mu_k, \Sigma_k)$. In model-based clustering the covariance matrix Σ_k determines the volume, shape and orientation of the components, which allows for different types of covariance structures to be modelled and compared [5].

Often, some of the covariance structures are unable to be estimated, due to singularity of the covariance matrix [5]. To overcome this issue, the Bayesian regularisation method, proposed by [7], was used in order to obtain an estimate for all covariance structures. This method replaces the maximum likelihood estimate by the maximum a posteriori estimate obtained from a Bayesian analysis. [7].

The optimal number of clusters and covariance parameterisation was selected by comparing the Bayesian Information Criterion (BIC). The BIC is a model-fit criterion which adds a penalty for models with increased complexity and is outlined as follows

$$BIC = 2l_{M,G}(y|\hat{\theta}) - vlog(n), \tag{3}$$

where $l_{M,G}(y|\hat{\theta})$ is the log-likelihood for the MLE $\hat{\theta}$ for model M with G clusters, n is the sample size and v is the number of estimated parameters [5]. A model is selected that minimises the BIC.

Results

The mclust package [5] in R was used to fit the model. This package allows for 14 different covariance patterns to be fit and compared using the BIC. Initially, all models were compared, however only 4 out of the 14 models were able to be estimated due to singularity issues with the covariance matrix. Therefore, Bayesian regularisation was employed, in order for more covariance structures to be compared. The default multivariate normal priors were used as described in [8]. After regularisation, 10 out of the 14 models were able to be compared. Figure 3 compares the BIC for the 10 different covariance structures specified at 2 to 20 clusters.

The best fitting model that minimized the BIC consists of 4 clusters and has a VII covariance structure. This refers to spherical clusters that have variable volume, but equal shape. In other words, the covariance between the functional domains are set to zero, indicating independence between the functional domains. The descriptive statistics for the four clusters are displayed in Table 2 and the cluster profiles for the four groups are displayed in Figure 4.

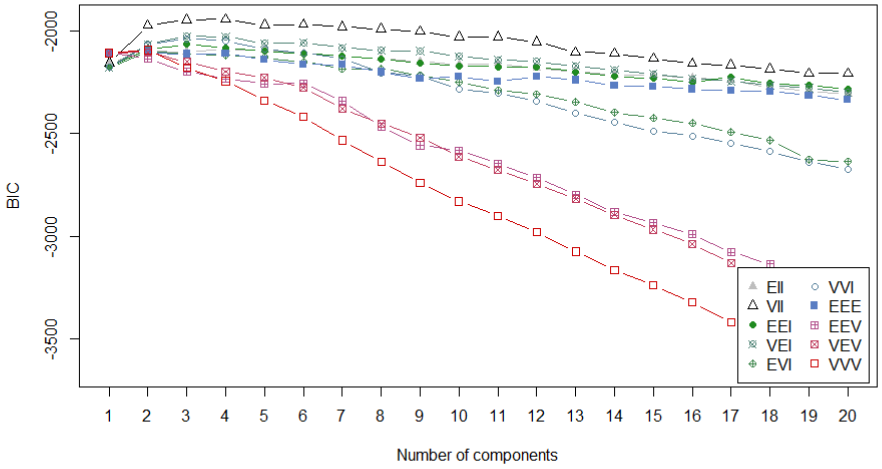

Figure 3: BIC for all covariance structures specified at 2 to 20 clusters.

It can be seen, in Figure 4, that Group 1 and Group 2 are similar to the two groups identified from the K-means, whereby Group 1 contains the majority of children, who display close to typical development across all functional domains and Group 2 is characterised by moderate to large deviations from typical development for all domains. The model-based clustering also identified two smaller clusters. Group 3 consists of 9 children and is characterised by larger deviations in the auditory, tactile and vision domains and typical development in the remaining domains. Group 4 contains 8 children who display typical development across all domains. Groups 3 and 4 are similar to groups 5 and 8 identified through the DPMM (see Figure 3 of the main paper).

Discussion

K-means and model-based clustering were used to cluster the areas obtained from the Bayesian sequential updating in order to compare the performance of the DPMM to these more commonly used clustering methods. The K-means clustering only returned two groups best characterised as typical and atypical development. The model-based clustering returned four groups. The first two groups were similar to the groups identified from the K-means, whereas the third and fourth group were smaller and were similar to groups that were identified using the DPMM. In contrast to these two alternative methods, the DPMM recovered nine groups (refer to Figure 3 of the main paper for a description of these groups).

Although K-means is a simple and fast algorithm to implement, it often

Table 2: Cluster sizes, means and standard deviations for the four clusters obtained from the model-based clustering

Cluster	Size	Domain	Mean	Standard Deviation
1	41	Auditory	0.025	0.045
		Hands	0.010	0.022
		Movement	0.020	0.050
		Speech	0.027	0.052
		Tactile	0.010	0.020
		Vision	0.007	0.011
2	21	Auditory	0.130	0.097
		Hands	0.075	0.116
		Movement	0.071	0.074
		Speech	0.202	0.164
		Tactile	0.077	0.084
		Vision	0.087	0.077
3	9	Auditory	0.098	0.077
		Hands	0.008	0.016
		Movement	0.008	0.001
		Speech	0.005	0.001
		Tactile	0.066	0.058
		Vision	0.119	0.110
4	8	Auditory	0.001	0.000
		Hands	$<$0.001	0.000
		Movement	$<$0.001	0.000
		Speech	0.001	$<$0.001
		Tactile	$<$0.001	0.000
		Vision	0.001	0.000

performs poorly when detecting clusters that have non-spherical shapes or differ significantly in size [9]. This explains why only two, roughly equal sized groups were optimal when using K-means. Furthermore, as the number of clusters k needs to be specified before running the analysis, it can often be difficult to select the optimal number of clusters. In order to be consistent with the DPMM, the average silhouette width method was used to select the optimal number of clusters, however, inspection of Figure 1 shows that there are a number of other cluster sizes that have average silhouette widths that are close to the optimal number, indicating that there may be other sensible alternative clustering solutions. In addition, the average silhouette method is only one index that can be used to find the optimal clusters. The Nbclust package [10] compares 30 indices for selecting the optimal number of clusters. When implementing all of these indices, only seven out of the 30 indices selected two clusters as the optimal number, providing further evidence that there may be alternative clustering solutions.

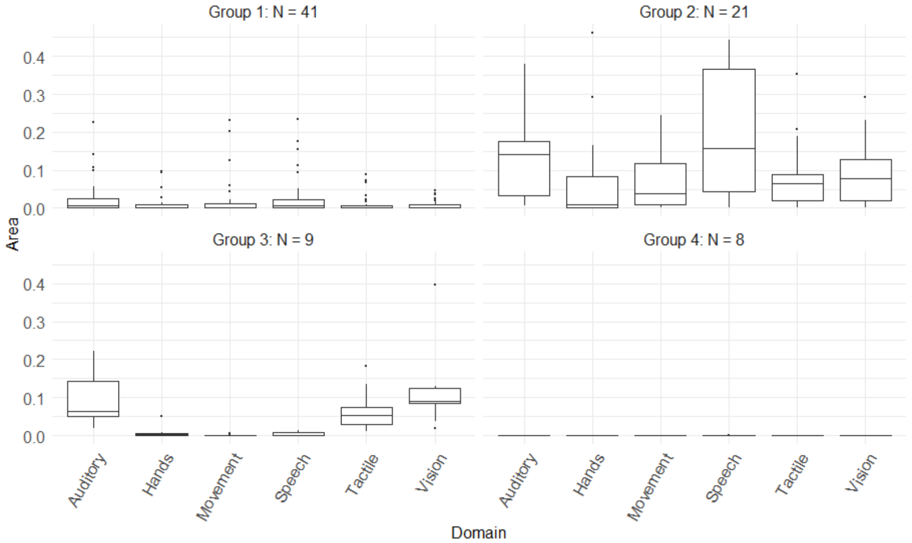

Figure 4: Cluster profiles for the 4 groups obtained using model-based clustering

The model-based clustering performed slightly better than K-means as it also identified two of the smaller clusters identified in the DPMM. Model-based clustering offers much more flexibility than K-means, in terms of the wide range of covariance structures that can be modelled [5]. However, despite this flexibility, it can fail to converge due to singularity in the covariance estimate, particularly for models with more complex covariance structures [7]. This was the case in the current implementation, where Bayesian regularisation needed to be used, in order to estimate more covariance structures. Estimating more covariance structures, however, also leads to difficulties with model selection. The BIC was used to select the best model, which has been known to favour simpler model structures [11]. This appears to be the case here, where models with spherical covariance structures resulted in lower BIC's. However, in Figure 3, it can be seen that there is not a lot of difference in BIC values between several competing covariance structures and number of clusters, suggesting that there may be several models that would provide sensible clustering solutions.

The DPMM retrieved more clusters than either of these alternative methods, due to the models tendency to cluster outlying individual's into smaller emerging clusters. Modelling the uncertainty in the number of clusters is a key feature of the DPMM, which is not possible with these alternative methods, due to needing to specify the number of clusters *a priori*. The DPMM is more complicated to implement, with priors, samplers and post-processing methods needing to be carefully considered. However, both K-means and model-based clustering also run into problems when identifying the optimal number of clusters from many candidate models. This selection process can be difficult and appears to be

a feature of unsupervised clustering in general, rather than a disadvantage of any specific clustering method. Overall, the DPMM was able to identify smaller, more distinct clusters than these alternative methods, which is important for the current application where identifying smaller groups for targeted intervention is the goal.

References

[1] R Core Team. R: A Language and Environment for Statistical Computing; 2018. Available from: `https://www.R-project.org/`.

[2] Hartigan JA, Wong MA. Algorithm AS 136: A k-means clustering algorithm. Journal of the Royal Statistical Society Series C (Applied Statistics). 1979;28(1):100–108.

[3] James G, Witten D, Hastie T, Tibshirani R. An introduction to statistical learning. vol. 112. Springer; 2013.

[4] Friedman J, Hastie T, Tibshirani R. The elements of statistical learning. vol. 1. Springer series in statistics New York, NY, USA:; 2001.

[5] Scrucca L, Fop M, Murphy TB, Raftery AE. mclust 5: Clustering, classification and density estimation using gaussian finite mixture models. The R journal. 2016;8(1):289.

[6] McLachlan G, Peel D. Finite mixture models, willey series in probability and statistics; 2000.

[7] Fraley C, Raftery AE. Bayesian regularization for normal mixture estimation and model-based clustering. Journal of classification. 2007;24(2):155–181.

[8] Fraley C, Raftery AE, Murphy TB, Scrucca L. mclust version 4 for R: normal mixture modeling for model-based clustering, classification, and density estimation. Technical report; 2012.

[9] Tan PN, Steinbach M, Kumar V, et al. Cluster analysis: basic concepts and algorithms. Introduction to data mining. 2006;8:487–568.

[10] Charrad M, Ghazzali N, Boiteau V, Niknafs A. NbClust Package: finding the relevant number of clusters in a dataset. J Stat Softw. 2012;.

[11] Weakliem DL. A critique of the Bayesian information criterion for model selection. Sociological Methods & Research. 1999;27(3):359–397.
