# Supplementary material for: Identifying latent subgroups of children with developmental delay using Bayesian sequential updating and Dirichlet process mixture modelling
Source: PLoS One. 2020 Jun 2;15(6):e0233542. doi: 10.1371/journal.pone.0233542 (PMC7266333; doi:10.1371/journal.pone.0233542)
Supplement: S4 Appendix — (PDF) [file pone.0233542.s005.pdf]

S4 Appendix. Sensitivity analysis results

Table 1: Hyperparameters used for the 15 models.

Model	N_0	c_0	C_0	α
1	0.01	2	Σ_y	Gamma(1,1)
2	0.01	3	$0.5\Sigma_y$	Gamma(2,2)
3	0.05	3	Σ_y	Gamma(1,1)
4	0.05	3	$0.5\Sigma_y$	Gamma(1,1)
5	0.05	2	Σ_y	Gamma(2,2)
6	0.05	3	$0.5\Sigma_y$	Gamma(2,2)
7	0.10	3	Σ_y	Gamma(1,1)
8	0.10	3	Σ_y	Gamma(2,2)
9	0.10	3	$0.75\Sigma_y$	Gamma(2,2)
10	0.20	3	$0.75\Sigma_y$	Gamma(1,1)
11	0.20	3	Σ_y	Gamma(2,2)
12	0.50	3	Σ_y	Gamma(1,1)
13	0.50	3	Σ_y	Gamma(2,2)
14	1.00	3	Σ_y	Gamma(1,1)
15	1.00	3	Σ_y	Gamma(2,2)

Table 2: Gelman Rubin (GR) statistic for K and α; the average silhouette width of the clusters produced from the PAM method when $k = 3$, and the percentage of correct classifications for simulated well separated, adjacent and overlapping clusters

Scenario	Model	GR K	GR α	Average silhouette width	Classification accuracy (%)
Well-separated	1	1.00	1.00	1.000	100.00
	2	1.00	1.00	0.999	100.00
	3	1.00	1.00	1.000	100.00
	4	1.00	1.00	0.998	100.00
	$5^{\dagger}$	1.00	1.00	1.000	100.00
	6	1.00	1.00	0.998	100.00
	7	1.00	1.00	0.999	100.00
	8	1.00	1.00	0.999	100.00
	9	1.00	1.00	0.999	100.00
	10	1.00	1.00	0.998	100.00
	11	1.00	1.00	0.999	100.00
	12	1.00	1.00	0.999	100.00
	13	1.00	1.00	0.999	100.00
	14	1.00	1.00	0.998	100.00
	15	1.00	1.00	0.998	100.00
Adjacent	1	1.00	1.00	0.948	100.00

Continued on next page

Table 2 – continued from previous page

Scenario	Model	GR K	GR α	Average silhouette width	Classification accuracy (%)
	2	1.00	1.00	0.931	100.00
	3	1.00	1.00	0.937	100.00
	4	1.00	1.00	0.869	100.00
	5	1.00	1.00	0.945	100.00
	6	1.00	1.00	0.869	100.00
	7	1.00	1.00	0.926	100.00
	8	1.00	1.00	0.927	100.00
	9	1.00	1.00	0.903	100.00
	10	1.00	1.00	0.887	100.00
	11	1.00	1.00	0.915	100.00
	12	1.00	1.00	0.907	100.00
	13	1.00	1.00	0.907	100.00
	14	1.00	1.00	0.907	100.00
	15	1.00	1.00	0.907	100.00
Overlapping	1	1.01	1.00	0.641‡	84.66
	2	1.00	1.00	0.622	76.67
	3	1.00	1.00	0.577‡	91.33
	4	1.00	1.00	0.493	78.67
	5	1.00	1.00	0.604‡	88.00
	6	1.00	1.00	0.491	78.67
	7	1.00	1.00	0.553	92.67
	8	1.00	1.00	0.554	93.33
	9	1.00	1.00	0.498	92.00
	10	1.00	1.00	0.475	92.00
	11	1.00	1.00	0.533	94.00
	12	1.00	1.00	0.523	93.33
	13	1.00	1.00	0.523	93.33
	14	1.00	1.00	0.522‡	92.67
	15	1.00	1.00	0.525‡	92.67

† Chain 3 failed to run, so results are based on Chain 1 and 2 only.

‡ For these models, 2 clusters corresponded to the maximum average silhouette width, the average silhouette width displayed here is for 3 clusters which was the second highest silhouette width for these models.

Traceplots for K and α, and screeplots for the average silhouette widths are available on Github [1]

Table 3: Gelman Rubin (GR) statistic for K and α; the average silhouette width of the clusters produced from the PAM method when $k = 3$, and the percentage of correct classifications for simulated small ($N = 150$), medium ($N = 1500$) and large sample sizes ($N = 15000$).

Scenario	Model	GR K	GR α	Average silhouette width	Classification accuracy (%)
Small	1	1.01	1.00	0.864	95.33
	2	1.03	1.01	0.798	95.33
	3	1.00	1.00	0.782	95.33
	4	1.01	1.00	0.630	95.33
	5	1.02	1.00	0.845	95.33
	6	1.02	1.01	0.623	95.33
	7	1.00	1.00	0.746	95.33
	8	1.00	1.00	0.740	95.33
	9	1.00	1.00	0.659	95.33
	10	1.00	1.00	0.600	95.33
	11	1.01	1.00	0.691	95.33
	12	1.00	1.00	0.659	95.33
	13	1.00	1.00	0.655	95.33
	14	1.00	1.00	0.674	95.33
	15	1.00	1.00	0.672	95.33
Medium	1	1.02	1.00	0.899	96.53
	2	1.09	1.01	0.876	96.53
	3	1.11	1.03	-	-
	4	1.07	1.03	0.761	96.40
	5	1.04	1.00	0.892	96.53
	6	1.04	1.02	0.771	96.47
	7	1.10	1.03	0.832	96.53
	8	1.01	1.00	0.817	96.47
	9	1.03	1.01	0.768	96.40
	10	1.06	1.04	0.701	96.40
	11	1.04	1.01	0.807	96.47
	12	1.05	1.02	0.793	96.47
	13	1.03	1.02	0.795	96.47
	14	1.01	1.00	0.838	96.53
	15	1.03	1.01	0.841	96.53
Large	1	1.25	1.00	-	-
	2	1.05	1.00	0.960	98.59
	3	1.10	1.01	0.964	98.59
	4	1.12	1.02	-	-
	5	1.05	1.00	0.959	98.58
	6	1.14	1.04	-	-

Continued on next page

Table 3 – continued from previous page

Scenario	Model	GR K	GR α	Average silhouette width	Classification accuracy (%)
	7	1.15	1.03	-	-
	8	1.15	1.04	-	-
	9	1.07	1.02	0.936	98.59
	10	1.11	1.06	-	-
	11	1.21	1.07	-	-
	12	1.04	1.01	0.934	98.60
	13	1.06	1.02	0.921	98.58
	14	1.07	1.02	0.929	98.57
	15	1.04	1.02	0.923	98.59

Traceplots for K and α and scree plots for the average silhoutte widths are available on Github [1]

References

[1] Gilholm P. Bayesian Sequential Updating DPMM supplementary; 2019. Available from: https://github.com/TrishG89/Bayesian_Sequential_Updating_DPMM_supplementary.
