# Supplementary material for: Identifying latent subgroups of children with developmental delay using Bayesian sequential updating and Dirichlet process mixture modelling
Source: PLoS One. 2020 Jun 2;15(6):e0233542. doi: 10.1371/journal.pone.0233542 (PMC7266333; doi:10.1371/journal.pone.0233542)
Supplement: Attachment — Submitted filename: Response to reviewers.docx [file pone.0233542.s007.docx]

**Response to reviewers
Manuscript PONE-D-19-29401**

**Identifying latent subgroups of children with developmental delay using Bayesian sequential updating and Dirichlet process mixture modelling**

A copy of our revised manuscript has been provided in the document labelled “Manuscript”. In addition, a copy of the manuscript with changes highlighted is also provided in the document labelled “Revised manuscript with track changes”. we have added additional documents to the first author’s Github, as requested. We have indicated where we have implemented the changes in each response to the reviewer’s comments; line numbers are those corresponding to the “Manuscript” document.

We would like to thank the Academic Editor and Reviewers for providing additional comments for this round of revisions. Following are our responses to the comments. Reviewers’ comments are italicised, with our response given immediately below the italicised text.

**Response to Academic Editor Comments**

*1. For transparency and replication, the authors are encouraged to prepare R codes (with sample data) for the three method sections: the Bayesian sequential updating, area between posterior probability sequences, and Dirichlet process mixture model and make them publicly available. Currently, only the slice sampling code is publicly available on Github.*

Thank you for this suggestion. We had R code for performing the Bayesian sequential updating and area calculation on the first author’s Github, but this was not referred to explicitly in the manuscript. In addition, we have prepared an R markdown walk through, using simulated data, of the analyses performed in the paper. We have added the following line to the manuscript directing the reader to the Github account, where they can find this information (Line 235):

“R code for performing the Bayesian sequential updating and area calculation, as well as an example of using this code on simulated data is available on Github.”
